# Factors Associated with Online Hate Acceptance: A Cross-National Six-Country Study among Young Adults

**DOI:** 10.3390/ijerph19010534

**Published:** 2022-01-04

**Authors:** Magdalena Celuch, Atte Oksanen, Pekka Räsänen, Matthew Costello, Catherine Blaya, Izabela Zych, Vicente J. Llorent, Ashley Reichelmann, James Hawdon

**Affiliations:** 1 Faculty of Social Sciences, Tampere University, 33014 Tampere, Finland; magdalena.celuch@tuni.fi; 2 Economic Sociology, Department of Social Research, University of Turku, 20500 Turku, Finland; pekras@utu.fi; 3 Department of Sociology, Anthropology & Criminal Justice, Clemson University, Clemson, SC 29643, USA; mjcoste@clemson.edu; 4 URMIS—The Migrations and Society Research Unit (CNRS 8245-IMR IRD 205), Université Côte d’Azur, 06046 Nice, France; catherine.blaya@univ-cotedazur.fr; 5 Department of Psychology, University of Córdoba, 14004 Córdoba, Spain; izych@uco.es; 6 Department of Education, University of Córdoba, 14004 Córdoba, Spain; vjllorent@uco.es; 7 Department of Sociology, Virginia Tech University, Blacksburg, VA 24061, USA; avr@vt.edu (A.R.); hawdonj@vt.edu (J.H.)

**Keywords:** online hate, Internet, young adults, empathy, social dominance orientation, cross-national

## Abstract

The Internet, specifically social media, is among the most common settings where young people encounter hate speech. Understanding their attitudes toward the phenomenon is crucial for combatting it because acceptance of such content could contribute to furthering the spread of hate speech as well as ideology contamination. The present study, theoretically grounded in the General Aggression Model (GAM), investigates factors associated with online hate acceptance among young adults. We collected survey data from participants aged 18–26 from six countries: Finland (*n* = 483), France (*n* = 907), Poland (*n* = 738), Spain (*n* = 739), the United Kingdom (*n* = 959), and the United States (*n* = 1052). Results based on linear regression modeling showed that acceptance of online hate was strongly associated with acceptance of violence in all samples. In addition, participants who admitted to producing online hate reported higher levels of acceptance of it. Moreover, association with social dominance orientation was found in most of the samples. Other sample-specific significant factors included participants’ experiences with the Internet and online hate, as well as empathy and institutional trust levels. Significant differences in online hate acceptance levels and the strength of its connections to individual factors were found between the countries. These results provide important insights into the phenomenon, demonstrating that online hate acceptance is part of a larger belief system and is influenced by cultural background, and, therefore, it cannot be analyzed or combatted in isolation from these factors.

## 1. Introduction

The Internet is both an important source of information and a space for social contact for many adolescents and young adults [1,2,3]. However, it is also a common setting of hate speech exposure among adults [4] and especially young people [5,6]. Online hate (i.e., cyberhate, online hate speech) is an expression of prejudice and hatred against a group of people based on a certain characteristic-e.g., religion, ethnic background, gender identity. Thus, even when targeting individuals, online hate can elicit harm upon a community [7,8].

Understanding what leads young people to accept online hate is essential for fighting hate speech and informing intervention. If online hate is commonly accepted, appropriate countermeasures-undertaken either by the victim or bystanders upon witnessing online hate-may be more difficult to establish. Therefore, the present study investigates the associations between online hate acceptance among young adults and a range of psychological and behavioral factors. In addition, we also stress the importance of international similarities and cross-cultural differences in understanding the phenomenon. Thus, our study includes four European Union countries representing Northern, Southern, Central, and Eastern Europe, as well as two major English-speaking countries-the United States and the United Kingdom. These countries share certain cultural commonalities, but they map also to different regions according to values and welfare [9,10].

The study is theoretically grounded in the General Aggression Model (GAM) [11,12]. GAM is a comprehensive model for understanding aggression that considers various types of circumstances and risk factors for violence. Most importantly for the current study, GAM highlights numerous individual and situational factors that lead to violent behavior and has been previously used in online hate research [13]. For instance, GAM predicts that viewing violent content leads to desensitization, namely the habituation of individual emotional reaction as a result of repeated exposure to a stimulus [14]. Results of past research on both offline and online hate speech align with these expectations, suggesting that exposure to hateful content increases acceptance toward it [6,13,15]. Thus, we expected to obtain similar results.

According to GAM, decreased affective empathy is one of the potential consequences of desensitization [11]. Empathy refers to the process of gaining emotional insight into the affective state of another person [16] and feeling concerned for the distress caused to the victim [17]. Affective empathy evokes the willingness to reduce victim’s suffering and facilitates pro-social behaviors [18,19], but its connection to online hate acceptance has not been the focus of previous research. Based on the established characteristics of the phenomenon, we expected affective empathy to be negatively connected to online hate acceptance.

GAM states that the effects of any external influence on an individual depend on many personal factors, including their personal history, attitudes, and beliefs. GAM names past victimization as one of the factors leading to aggressive behaviors [11]. In the online hate context this expectation is supported by past research on children and adolescents that connects online victimization and perpetration [20] and names revenge as one of the strongest motivations for engaging in various bullying behaviors [21]. Unsurprisingly, more frequent use of hate speech has also been found to associate with lower desire to ban it, lower ratings of its offensiveness [15], and a greater tendency to agree with it [22]. Therefore, we expected both online hate victimization and online hate production to be positively associated with online hate acceptance. Moreover, demographic characteristics may be connected to online hate acceptance as well. For example, past research indicates that women are less likely to accept hate speech against minorities they tend to view it as more offensive and are more likely to support outlawing it [6,23].

Social dominance orientation (SDO) is another potentially relevant factor in online hate acceptance. SDO is defined as an individual’s attitude toward inequalities between social groups. Social-dominance-oriented individuals believe some social groups are inherently better than others and thus favor hierarchical organization of societies [24]. SDO has been previously associated with using hate speech [6] and disapproval of a ban on hate speech [25]. We therefore assumed that social-dominance oriented individuals will be more likely to accept online hate.

Trust in public institutions is another factor that may affect acceptance of online hate. Institutional trust refers to a belief in the credibility, fairness, competence, and transparency of state, political and social institutions [26,27,28]. It is correlated with various pro-social phenomena, including higher subjective well-being [29,30] and increased generalized trust [28,30,31]. Generalized trust in others, on a macro level, has been connected with numerous societal benefits, such as less dishonest behavior [32] and more cooperation between democratic governments and citizens [33]. Considering online hate, low levels of institutional trust have been previously connected to online hate exposure [34] and production [35]. Thus, we expected that institutional trust would be connected to online hate acceptance.

Finally, GAM states that social context can constitute an important factor for an individual’s behavior [11]. On many social media sites, algorithms are used to tailor the displayed content to fit the user’s interests. One possible result of such practices is the formation of close-knit online networks-or identity bubbles-that strengthen users’ existing views, while simultaneously reinforcing their separation from different groups and their potentially challenging perspectives [2]. The formation of online identity bubbles and subsequent collisions between them have been named as the driving forces of polarization and negativity in the online environment [36]. It is thus possible that individuals strongly involved in online communities find online hate more acceptable as a means to defend their group and its worldviews, and as a result, their group norms.

To summarize, this study investigated social and psychological factors associated with online hate acceptance among young adults. Our study was theoretically grounded in the General Aggression Model [11,12]. GAM has been broadly used to explain antisocial behaviors in general. There are reasons to believe that it could be applied to online hate acceptance. Based on the theory and past research on offline and online hate acceptance, we expected to find significant connections of online hate acceptance with participants’ individual history concerning online hate and Internet use, as well as a range of personal beliefs and attitudes. We were also interested in international similarities and cross-cultural differences in understanding the phenomenon. Thus, our research questions were:

RQ1: What factors associate with acceptance of online hate among young adults?

H1: Frequency of seeing hate material online is connected to online hate acceptance;

H2: Affective empathy is negatively related to online hate acceptance;

H3: Individual history with online hate, namely, (a) previous victimization and (b) online hate production, is connected to online hate acceptance;

H4: Social dominance orientation is connected to online hate acceptance;

H5: Institutional trust levels influence online hate acceptance;

H6: Involvement in online social networks is connected to online hate acceptance.

RQ2: Do these associations vary between Finland, France, Poland, Spain, the United Kingdom, and the United States?

## 2. Materials and Methods

### 2.1. Participants and Procedure

This study included young adults aged 18 to 26 years old from six countries. We collected data from Finland (*n* = 483), France (*n* = 907), Poland (*n* = 738), Spain (*n* = 739), the United Kingdom (*n* = 959), and the United States (*n* = 1052). The participants were recruited from the participant panel administrated by Dynata (formerly known as Survey Sampling International, see dynata.com). Data collection was carried out in May 2018. All groups received identical surveys in the majority language of their country. The surveys were originally constructed in English and then translated to the respective country’s majority language by researchers fluent in English and native speakers of the specific language. The surveys were then reviewed by additional native speakers. Previously validated measures were used in the surveys. The median response time for the survey varied between samples from 6 min 53 s in the United Kingdom sample to 10 min 28 s in the Polish sample. Participants were informed of the study objectives, assured their participation was voluntary and their answers were anonymous, and told they can resign from participating at any moment without any consequences. They were also provided contact information for one of the study’s principal investigators. An institutional review board statement was received from Virginia Tech in 2018 prior the data collection.

Due to overrepresentation of females in all samples (range = 58.65–71.08%), probability and analytic weights were used for analyses. Data concerning the percentage of females in each of the nations for the 15-to-25-year-old age group were used for the creation of weight variables [37]. Average age of the participants was similar in all samples, ranging from M = 21.42 (SD = 2.28) to M = 21.88 (SD = 2.21) years of age (see Table 1).

### 2.2. Measures

Acceptance of online hate. This variable was intended to assess participants’ views on how acceptable it is to send hateful and degrading online messages against someone. It was measured with five questions adapted from an acceptance of violence scale used in previous studies [38] to reflect acceptance toward online hate. The adapted version reflects acceptance of online hate and has been utilized in past research on the topic [39]. Items included are, “It is OK to send hateful or degrading messages against someone online if they start to attack you first” and, “It is OK to send hateful or degrading messages against someone online because this is how people respect you.” All items can be found in Appendix A. Participants assessed the degree to which they agreed with each of the statements on a 5-point Likert scale from 1 (strongly agree) to 5 (strongly disagree). The scale was later reversed so that higher results reflect greater acceptance of online hate. The scale had high reliability in all samples (McDonald’s ω ranging from 0.86 to 0.90).

Daily Internet use. The amount of daily Internet use was assessed with a single question: “Approximately how many hours per day do you use the Internet?” The response options ranged from 1 (less than 1 h per day) to 6 (10 or more hours a day).

Online hate exposure. Frequency of exposure to online hate material was measured with a single item: “How frequently do you see material online that expresses negative views toward some group?” Participants were asked to respond using a 4-point Likert scale ranging from 1 (I never see this type of material) to 4 (I see this type of material frequently).

Online hate victimization. To assess recent online hate victimization, participants were asked to indicate on a scale from 1 (never) to 5 (11 or more times) how often in the preceding 3 months they had been attacked based on nine characteristics, including their ethnicity, religion, political views, gender, and appearance. A dummy variable was then created to reflect recent online hate victimization (0 = no recent victimization, 1 = some recent victimization). Participants who reported being attacked at least once for any of the listed reasons were classified as recent online hate victims.

Online hate production. Participants were asked, “In the past 3 months, have you produced hateful or degrading writings or speech online that attacked certain groups of people or individuals?” and the responses were coded 0 = no and 1 = yes.

Sense of belonging to an online community. A single item with a 5-point response scale (1 = not at all close to 5 = very close) was used to measure how close participants felt to an online community to which they belonged.

Institutional trust. Five questions measured participants’ trust in various institutions and groups, namely the police, schools, colleges and universities, politicians, and mainstream media sources. For each of the questions, participants rated their trust on a 10-point scale ranging from 1 (you can’t be too careful) to 10 (can be fully trusted). Similar measures of trust toward various groups and institutions have been utilized in past research [27,28]. A scale comprised of these items had high reliability in all samples (McDonald’s ω ranging from 0.77 to 0.86).

Acceptance of violence. Acceptance of violence was measured using a 15-item scale that included statements adapted from past studies considering legal cynicism [40], moral disengagement [41], and pro-violence attitudes [38,39]. The full list of items is included in Appendix B. The items utilized 5-point Likert scale response options ranging from 1 (strongly agree) to 5 (strongly disagree). The scale was then reversed so that higher scores reflect greater acceptance of violence. The scale had excellent reliability in all samples (McDonald’s ω ranging from 0.86 to 0.91).

Social dominance orientation. Social dominance orientation was measured with 13-items from the Social Dominance Orientation Scale assessing individuals’ preferences for the degree of inequality among social groups [24]. The scale had excellent reliability in all samples (McDonald’s ω ranging from 0.85 to 0.90).

Empathy. Empathy was measured using the 16-item Toronto Empathy Questionnaire (TEQ). TEQ treats empathy as a primarily emotional process. It has been extensively validated and correlates strongly with other related self-reporting and behavioral measures [16]. The scale had very good reliability in all samples (McDonald’s ω ranging from 0.83 to 0.88).

### 2.3. Statistical Analyses

All analyses were performed using Stata 16.1 software (StataCorp LLC, College Station, TX, USA). We recorded a relatively high proportion of random missing answers with around 20% of participants in each sample having skipped at least one item in the survey. However, no patterns in the missing data were identified. For this reason, average means were calculated from each of the scales for each participant. These average means were then used for further analyses. Descriptive statistics of these recalculated variables are presented in Table 1 along with McDonald’s ω values recorded for each of the original scales. Use of McDonald’s ω has been recommended as a more optimal measure of reliability of multi-item scales compared to Cronbach’s α [42].

Linear regression modeling was used for answering the main research questions considering the factors associated with online hate acceptance and cross-country differences. We report six models for each of the six country samples, respectively, as well as additional analyses of cross-country differences. Collinearity diagnostics did not indicate any problems with multicollinearity in the data (VIF values < 2) [43]. However, heteroscedasticity of residuals was detected based on a graphic representation of the data; therefore, robust standard errors were reported in addition to the *p* values, 95% confidence intervals (CIs), and β values.

## 3. Results

### 3.1. Online Hate Victimization and Production Rates

After adjusting for the gender distribution in the data, the highest rate of online hate victimization was found among the U.S. sample, where 72.95% of participants reported experiencing at least one attack online during the previous 3 months. The lowest—but still rather high—proportion of victims (43.33%) was recorded in the Finnish sample. The percentage of young adults admitting to having ever produced hateful or degrading material ranged from 8.78% in the Polish sample to 19.29% in the United States. The rates for each of the samples are presented in Table 1.

### 3.2. Factors Associated with Online Hate Acceptance

#### 3.2.1. Finland

A multiple linear regression was calculated using data from 483 participants from Finland. A significant regression equation was found, *F*(11,471) = 63.53, *p* < 0.001, with an *R*^2^ of 0.59. Higher scores on the acceptance of violence scale (β = 0.48, *p* < 0.001) and the Social Dominance Orientation Scale (β = 0.16, *p* < 0.001), as well as sense of belonging to an online community (β = 0.11, *p* = 0.004), were significantly associated with acceptance of online hate. Online hate producers were also more likely to accept online hate (β = 0.10, *p* = 0.021). Institutional trust (β = −0.11, *p* = 0.003), Toronto Empathy Questionnaire scores (β = −0.11, *p* = 0.010), and identifying as female (β = −0.07, *p* = 0.043) were associated with the opposite effect. The remaining factors were not significant. The full model is outlined in Table 2.

#### 3.2.2. France

Data from 907 participants from France were utilized in a multiple linear regression analysis. A significant regression equation was found, *F*(11,895) = 56.95, *p* < 0.001, with an *R*^2^ of 0.44. Higher scores on acceptance of violence (β = 0.55, *p* < 0.001) and social dominance orientation (β = 0.11, *p* = 0.001) scales, as well as online hate production (β = 0.15, *p* < 0.001), were associated with greater acceptance of online hate. Institutional trust scores had a similar effect (β = 0.07, *p* = 0.029). An inverse relationship was found between online hate acceptance and the amount of time spent daily on the Internet (β = −0.09, *p* = 0.003), as well as age (β = −0.08, *p* = 0.005). The remaining factors were not significant in this model.

#### 3.2.3. Poland

A multiple linear regression was calculated using data from 738 participants from Poland. A significant regression equation was found, *F*(11,726) = 43.52, *p* < 0.001, with an *R*^2^ of 0.39. Acceptance of violence scores (β = 0.48, *p* < 0.001) and online hate production (β = 0.12, *p* = 0.001) were positively associated with online hate acceptance, whereas a negative association was found for identifying as female (β = −0.07, *p* = 0.048). The remaining factors did not produce significant results in this model.

#### 3.2.4. Spain

Data from 739 participants from Spain were included in a multiple linear regression model, for which a significant regression equation was found, *F*(11,727) = 32.11, *p* < 0.001 with an *R*^2^ of 0.41. Similar to previous models, online hate production (β = 0.15, *p* < 0.001), acceptance of violence (β = 0.46, *p* < 0.001), and social dominance orientation scores (β = 0.13, *p* = 0.001) were positively associated with online hate acceptance. Moreover, in this sample, frequency of exposure to online hate was also positively connected to its acceptance (β = 0.08, *p* = 0.011). Other factors included in the model were not significant.

#### 3.2.5. United Kingdom

Data from 959 participants from the United Kingdom were utilized in a multiple linear regression analysis. A significant regression equation was found, *F*(11,947) = 74.10, *p* < 0.001, with an *R*^2^ of 0.47. Once again, online hate production (β = 0.13, *p* < 0.001) and acceptance of violence scores (β = 0.49, *p* < 0.001) were associated with online hate acceptance. Moreover, higher Toronto Empathy Questionnaire scores (β = −0.12, *p* = 0.005) were connected to lower acceptance for online hate. No other significant relationships were found.

#### 3.2.6. United States

A multiple linear regression was calculated using data from 1052 participants from the United States. A significant regression equation was found, *F*(11,1040) = 120.85, *p* < 0.001, with an *R*^2^ of 0.57. Acceptance of violence (β = 0.51, *p* < 0.001) and social dominance orientation scores (β = 0.13, *p* < 0.001), as well as online hate production (β = 0.16, *p* < 0.001), were positively related to online hate acceptance. Online hate victimization in the preceding 3 months had a similar effect (β = 0.04, *p* = 0.047). Finally, identifying as female was negatively connected to online hate acceptance (β = −0.07, *p* = 0.003).

### 3.3. Cross-Country Differences

A single linear regression model (*n* = 4878) was calculated to check for statistical differences in online hate acceptance levels between countries. Changing the reference category between the countries allowed for detailed comparisons. A significant regression equation was found, *F*(5,4872) = 5.77, *p* < 0.001, with an *R*^2^ of 0.01, meaning that levels of online hate acceptance differed significantly between the countries. The highest rate of online hate acceptance was recorded in the United States, where it was significantly higher than it was in Finland (*t* = −2.54, *p* = 0.011), France (*t* = −2.61, *p* = 0.009), and Spain (*t* = −4.95, *p* < 0.001). The lowest online hate acceptance was recorded in Spain, where it was significantly lower than it was in France (*t* = 2.63, *p* = 0.008), Poland (*t* = 3.34, *p* = 0.001), the United Kingdom (*t* = 3.80, *p* < 0.001) and the United States (*t* = 4.95, *p* < 0.001). No significant differences were found between the other countries.

Further country differences were analyzed with country interactions in the full linear model (*n* = 4878). The association between online hate acceptance and acceptance of violence was stronger in Finland than it was in Spain (β = −0.15, *p* = 0.019) and the United Kingdom (β = −0.14, *p* = 0.024). Social dominance orientation scale scores had more impact on online hate acceptance in Finland than in France (β = −0.12, *p* = 0.011) and Spain (β = −0.09, *p* = 0.034). Finally, the effect of institutional trust was stronger in France than it was in Finland (β = −0.15, *p* = 0.001).

## 4. Discussion

The present research investigated factors associated with online hate acceptance among young adults. We found that online hate acceptance was associated with various individual attitudes and behaviors in all samples. Thus, most of our expectations, based on previous research and the GAM [11,12], were at least partially confirmed. Moreover, we found many cross-national similarities, but also multiple differences in online hate acceptance levels, associated factors, and strengths of these correlations.

According to results, online hate acceptance was connected to general pro-violence attitudes in all six samples. This is an important outcome because it suggests that approving of online hate is a part of a larger belief system. This conclusion is further strengthened by the results showing that higher social dominance orientation was positively related to online hate acceptance in four of our samples. These results demonstrate that acceptance of online hate does not occur in a vacuum, but is strongly interconnected with other attitudes-namely, pro-violence and pro-social inequalities beliefs.

Participants’ experiences with online hate and the Internet overall also predicted online hate acceptance. Most notably, in all six samples, online hate producers were more likely to find online hate acceptable. Based on our data, it is not possible to determine the direction of this association, but it is likely that young adults post hateful messages online because they deem it an acceptable behavior. However, it is also possible that online hate acceptance develops afterwards, as a way of justifying that behavior. Further studies on the matter are needed to clarify this issue.

Other Internet-related behaviors and attitudes were also significant predictors of online hate acceptance in some of our samples. Online hate acceptance was negatively associated with frequency of daily Internet use among French participants. It was also positively related to online hate exposure in the Spanish sample, and to past online hate victimization among participants from the United States. In the Finnish sample, participants who felt a stronger sense of belonging to an online community were more likely to find online hate acceptable. These results point to the importance of everyday experiences with the Internet and social media for online hate acceptance. Importantly, these conclusions are in line with past research and theoretical assumptions concerning the negative impact of online hate exposure and victimization on acceptance of the phenomenon [11,13,15]. However, our results were not consistent between the samples. This is possibly due to the intricate nature of these connections. For instance, past research suggests that the relationships between individuals’ experiences with online hate and their later behavior are moderated by various factors [20,44] and that previous victimization can be, somewhat in contrast to our assumptions and results, a motivation for defending others [45]. Therefore, it is possible that the existence and direction of these connections depends on a range of factors beyond the scope of this study. A closer investigation of these relations would be needed to clarify the matter.

Empathy had the expected negative relation to online hate acceptance in two samples; namely, participants from Finland and the United Kingdom. The relation was not significant in other samples. However, we measured participants’ declared empathy levels and we focused on affective empathy. Past research suggests that a cognitive empathy induction intervention can decrease online aggression and increase the chance of reporting abusive content [46,47,48]. Therefore, it is possible that an intervention would yield significant results concerning online hate acceptance, even when existing levels of empathy produced only negligible outcomes.

We found contradictory results concerning the impact of institutional trust. While it was not significant in most of our samples, it had a positive relationship with online hate acceptance in the French sample, and a negative effect in the Finnish sample. These and other differences between the countries can stem from multiple sources, including certain country-specific differences, such as differences in legislation or varying political and social systems, which may impact, for instance, who deems certain institutions as trustworthy and the extent to which they do so.

Finally, females were less likely to report acceptance of online hate in half of our samples-Finland, Poland, and the United States. These gender differences coincide with many studies about antisocial behaviors [49].

Considering cross-country differences, online hate acceptance levels were significantly higher in the United States, and significantly lower in Spain, than they were in most of our samples. The extent to which pro-violence attitudes, social dominance orientation, and institutional trust affected online hate acceptance also differed between some of the samples. Overall, these observations show that even though our key findings were constant across the samples, some specific variations did occur. We believe these can be attributed to overall cultural differences. Indeed, the countries included in this study, despite many cultural similarities, belong to different regions in terms of values and welfare [9,10]. Our results show that although many key principles seem to transcend country borders, awareness of cultural context is also essential in understanding online hate acceptance.

The current research has certain limitations. The cross-sectional nature of the data does not allow for establishing causal relationships. Future research using longitudinal design could expand the current results by observing the development of online hate acceptance. Moreover, our samples included emerging adults from Western countries. Other cultural spheres could be included in future studies.

Despite these limitations, the present research carries important theoretical and practical implications. This study was theoretically grounded in the GAM [11,12]. Although the primary goal of GAM is to explain aggressive behavior, the results of the present study demonstrate that its assumptions can be successfully applied to violence-related attitudes, specifically to online hate acceptance. Further exploration of GAM’s usefulness for online hate research is an interesting avenue for future research. Concerning practitioners, the results of this study situate online hate acceptance in a broader context of pro-violence and pro-inequality attitudes. This suggests educational efforts need to consider the broader worldviews held by their targeted audiences, as well as their cultural context and previous experiences with the Internet and online social networks. This could mean, for instance, inquiring about these experiences and beliefs while working with individuals, and, in group-based programs, combining discussion on online hate with related societal interventions.

## 5. Conclusions

The present research investigated factors associated with online hate acceptance among young adults from six countries (Finland, France, Poland, Spain, the United Kingdom, and the United States) using assumptions derived from the GAM. We recorded cross-country differences in online hate acceptance levels. We found that online hate acceptance was connected to violence acceptance and online hate production in all samples. Social dominance orientation was associated with online hate acceptance in most samples. Participants’ experiences with the Internet and online social networks, along with other sample-specific factors, also predicted online hate acceptance in some samples. The level of influence that some of these factors had on online hate acceptance differed between the countries. These results advance our understanding of who finds online hate acceptable and why they do so by placing online hate acceptance in a broader context of violence research and pointing toward individuals’ worldviews, online behaviors, and cultural backgrounds as crucial factors for both future research and practice.

## Figures and Tables

**Table 1:** Descriptive statistics.

	Finland				France				Poland			
**Categorical Variables**	***n***	**%**			***n***	**%**			***n***	**%**		
Online hate victims	209	43.33			489	53.87			479	64.88		
Online hate producers	86	17.87			85	9.33			65	8.78		
Gender												
Male	242	50.14			453	49.91			377	51.06		
Female	241	49.86			454	50.09			361	48.94		
**Continuous Variables**	**Range**	***M***	***SD***	**ω**	**Range**	***M***	***SD***	**Ω**	**Range**	***M***	***SD***	**ω**
Acceptance of online hate	1–5	1.99	0.94	0.86	1–5	2.01	0.95	0.86	1–5	2.06	0.98	0.86
Daily Internet use	1–6	3.46	1.18		1–6	3.37	1.24		1–6	3.67	1.21	
Frequency of seeing hate material online	1–4	2.89	0.76		1–4	2.61	0.87		1–4	2.76	0.74	
Belonging to online community	1–5	2.55	1.11		1–5	2.42	1.18		1–5	2.47	1.00	
Institutional trust	1–10	6.06	1.84	0.86	1–10	5.04	1.78	0.82	1–10	4.51	1.59	0.77
Acceptance of violence	1–5	2.19	0.58	0.87	1–5	2.35	0.63	0.86	1–5	2.36	0.64	0.87
Social dominance orientation	1–6.54	2.81	1.08	0.90	1–5.33	2.87	1.03	0.87	1–5.54	2.98	0.94	0.85
Empathy	1.69–5	3.66	0.58	0.88	2–5	3.65	0.60	0.83	2.06–5	3.59	0.53	0.83
Age	18–26	21.43	2.31		18–26	21.46	2.20		18–26	21.42	2.28	
	**Spain**				**United Kingdom**				**United States**			
**Categorical Variables**	***n***	**%**			***n***	**%**			***n***	**%**		
Online hate victims	484	65.54			605	63.07			767	72.95		
Online hate producers	80	10.86			115	12.01			203	19.29		
Gender												
Male	381	51.53			485	50.60			522	49.61		
Female	358	48.47			474	49.40			530	50.39		
**Continuous variables**	**Range**	***M***	***SD***	**ω**	**Range**	***M***	***SD***	**ω**	**Range**	***M***	***SD***	**ω**
Acceptance of online hate	1–5	1.88	0.96	0.89	1–5	2.08	1.04	0.89	1–5	2.15	1.13	0.90
Daily Internet use	1–6	3.72	1.23		1–6	3.91	1.32		1–6	3.93	1.44	
Frequency of seeing hate material online	1–4	2.50	0.94		1–4	2.60	0.88		1–4	2.75	0.91	
Belonging to online community	1–5	2.65	1.19		1–5	2.63	1.18		1–5	2.81	1.26	
Institutional trust	1–10	5.18	1.69	0.77	1–10	5.24	1.82	0.81	1–10	5.37	1.87	0.81
Acceptance of violence	1–5	2.20	0.65	0.89	1–5	2.33	0.74	0.90	1–5	2.38	0.81	0.91
Social dominance orientation	1–5.92	2.73	1.04	0.87	1–6.08	2.86	1.12	0.89	1–7	2.85	1.18	0.90
Empathy	1.94–5	3.76	0.59	0.87	2–5	3.58	0.58	0.84	2.19–5	3.63	0.62	0.86
Age	18–26	21.83	2.25		18–26	21.69	2.27		18–26	21.88	2.21	

**Table 2:** Linear regression models for acceptance of online hate.

	Finland							France						
**Acceptance of Online Hate**	***B***	***SE* (*B*)**	***t***	***p***	**95% CI (*B*)**		**β**	***B***	***SE* (*B*)**	***t***	***p***	**95% CI (*B*)**		**β**
Daily Internet use	−0.04	0.03	−1.36	0.174	−0.09	0.02	−0.05	−0.07	0.02	−2.95	0.003	−0.11	−0.02	−0.09
Frequency of online hate exposure	0.08	0.04	1.96	0.051	0.00	0.17	0.07	0.05	0.03	1.61	0.108	−0.01	0.11	0.05
Online hate victimization	0.07	0.06	1.03	0.305	−0.06	0.19	0.03	0.08	0.05	1.58	0.115	−0.02	0.19	0.04
Online hate production	0.24	0.10	2.31	0.021	0.04	0.44	0.10	0.50	0.10	5.02	<0.001	0.31	0.70	0.15
Sense of belonging to an online community	0.09	0.03	2.91	0.004	0.03	0.15	0.11	0.00	0.02	0.07	0.947	−0.04	0.05	0.00
Institutional trust	−0.06	0.02	−2.99	0.003	−0.09	−0.02	−0.11	0.04	0.02	2.19	0.029	0.00	0.07	0.07
Acceptance of violence	0.77	0.07	10.98	<0.001	0.64	0.91	0.48	0.83	0.06	13.34	<0.001	0.70	0.95	0.55
Social dominance orientation	0.14	0.04	3.63	<0.001	0.07	0.22	0.16	0.10	0.03	3.32	0.001	0.04	0.16	0.11
Empathy	−0.19	0.07	−2.58	0.010	−0.33	−0.04	−0.11	0.01	0.06	0.20	0.840	−0.10	0.12	0.01
Gender (female)	−0.14	0.07	−2.03	0.043	−0.27	0.00	−0.07	0.00	0.05	−0.05	0.957	−0.11	0.10	0.00
Age	0.02	0.01	1.43	0.154	−0.01	0.05	0.05	−0.03	0.01	−2.83	0.005	−0.06	−0.01	−0.08
Constant	4.77	0.48	9.87	<0.001	3.82	5.72		5.22	0.44	11.80	<0.001	4.36	6.09	
	*n*		483					*n*		907				
	*F*(11,471)		63.53					*F*(11,895)		56.95				
	*p*		<0.001					*p*		<0.001				
	*R* ^2^		0.59					*R* ^2^		0.44				
	**Poland**							**Spain**						
**Acceptance of Online Hate**	***B***	***SE* (*B*)**	***t***	***p***	**95% CI (*B*)**		**β**	***B***	***SE* (*B*)**	***T***	***p***	**95% CI (*B*)**		**β**
Daily Internet use	0.05	0.03	1.86	0.063	0.00	0.10	0.06	0.02	0.02	0.80	0.422	−0.03	0.06	0.02
Frequency of online hate exposure	−0.09	0.05	−1.72	0.086	−0.20	0.01	−0.07	0.08	0.03	2.55	0.011	0.02	0.14	0.08
Online hate victimization	0.11	0.07	1.65	0.099	−0.02	0.24	0.05	0.07	0.06	1.23	0.221	−0.04	0.19	0.04
Online hate production	0.42	0.13	3.37	0.001	0.18	0.67	0.12	0.47	0.11	4.13	<0.001	0.24	0.69	0.15
Sense of belonging to an online community	0.03	0.04	0.79	0.432	−0.04	0.10	0.03	0.01	0.03	0.49	0.624	−0.04	0.06	0.02
Institutional trust	−0.03	0.02	−1.37	0.170	−0.08	0.01	−0.05	0.01	0.02	0.69	0.491	−0.03	0.05	0.02
Acceptance of violence	0.73	0.07	10.72	<0.001	0.60	0.87	0.48	0.67	0.07	9.04	<0.001	0.52	0.81	0.46
Social dominance orientation	0.06	0.04	1.64	0.101	−0.01	0.14	0.06	0.12	0.04	3.29	0.001	0.05	0.19	0.13
Empathy	−0.08	0.08	−0.99	0.323	−0.23	0.08	−0.04	−0.10	0.07	−1.33	0.185	−0.24	0.05	−0.06
Gender (female)	−0.13	0.07	−1.98	0.048	−0.27	0.00	−0.07	−0.09	0.06	−1.43	0.152	−0.20	0.03	−0.04
Age	−0.01	0.01	−0.48	0.629	−0.04	0.02	−0.02	−0.01	0.01	−0.81	0.416	−0.03	0.01	−0.02
Constant	5.09	0.49	10.39	<0.001	4.13	6.05		4.25	0.51	8.40	<0.001	3.26	5.25	
	*n*		738					*n*		739				
	*F*(11,726)		43.52					*F*(11,727)		32.11				
	*p*		<0.001					*p*		<0.001				
	*R* ^2^		0.39					*R* ^2^		0.41				
	**United Kingdom**							**United States**						
**Acceptance of Online Hate**	***B***	***SE* (*B*)**	***t***	***p***	**95% CI (*B*)**		**β**	***B***	***SE* (*B*)**	***T***	***p***	**95% CI (*B*)**		**β**
Daily Internet use	−0.02	0.02	−0.95	0.343	−0.07	0.02	−0.03	−0.02	0.02	−1.34	0.182	−0.06	0.01	−0.03
Frequency of online hate exposure	0.02	0.04	0.43	0.670	−0.06	0.09	0.01	0.00	0.03	−0.08	0.939	−0.07	0.06	0.00
Online hate victimization	0.07	0.06	1.10	0.273	−0.05	0.18	0.03	0.11	0.06	1.99	0.047	0.00	0.22	0.04
Online hate production	0.41	0.11	3.58	<0.001	0.18	0.63	0.13	0.47	0.09	5.42	<0.001	0.30	0.64	0.16
Sense of belonging to an online community	0.03	0.03	1.21	0.226	−0.02	0.08	0.04	0.03	0.02	1.36	0.176	−0.01	0.07	0.03
Institutional trust	−0.03	0.02	−1.41	0.158	−0.06	0.01	−0.05	−0.01	0.01	−0.66	0.511	−0.04	0.02	−0.02
Acceptance of violence	0.69	0.07	10.42	<0.001	0.56	0.82	0.49	0.71	0.04	16.98	<0.001	0.63	0.80	0.51
Social dominance orientation	0.06	0.04	1.55	0.121	−0.01	0.13	0.06	0.12	0.03	3.57	<0.001	0.05	0.19	0.13
Empathy	−0.22	0.08	−2.78	0.005	−0.37	−0.06	−0.12	−0.09	0.06	−1.38	0.167	−0.21	0.04	−0.05
Gender (female)	−0.04	0.06	−0.71	0.475	−0.16	0.07	−0.02	−0.16	0.05	−3.02	0.003	−0.27	−0.06	−0.07
Age	−0.01	0.01	−0.40	0.687	−0.03	0.02	−0.01	0.00	0.01	0.27	0.786	−0.02	0.03	0.01
Constant	5.39	0.46	11.67	<0.001	4.49	6.30		4.62	0.43	10.69	<0.001	3.78	5.47	.
	*N*		959					*n*		1052				
	*F*(11, 947)		74.10					*F*(11,1040)		120.85				
	*p*		<0.001					*p*		<0.001				
	*R* ^2^		0.47					*R* ^2^		0.57				

## Data Availability

Data will available from authors with a reasonable request.

## Appendix A

### Online Hate Acceptance

Now, thinking about hateful or degrading messages and comments on the internet (e.g., insulting someone on social media), how strongly do you agree or disagree with the following statements?

1. It is OK to send hateful or degrading messages against someone online if they start to attack you first.
2. It is OK to send hateful or degrading messages against someone online if they insult your friends or your family.
3. It is OK to send hateful or degrading messages against other people online because hate speech is fun.
4. It is OK to send hateful or degrading messages against someone online because this is how people respect you.
5. It is OK to send hateful or degrading messages against someone online if these people make fun of you or insult you because of your religion, your origin, or the color of your skin.

## Appendix B

### Acceptance of Violence

Please indicate if you strongly agree, agree, neither agree nor disagree, disagree, or strongly disagree with each of the following statements.

1. Laws are made to be broken.
2. It is okay to do anything you want as long as you don’t hurt anyone.
3. Fighting between friends or within families is nobody else’s business.
4. It is alright to lie to keep your friends out of trouble.
5. Damaging some property is no big deal when you consider that others are beating people up.
6. Compared to the illegal things people do, taking some things from a store without paying for them is not very serious.
7. Kids cannot be blamed for misbehaving if their friends pressured them to do it.
8. It is OK to use violence against someone if they start to fight with you first.
9. It is OK to use violence against someone if they insult your friends or your family.
10. It is OK to use violence because violence is fun.
11. It is OK to use violence because this is how people respect you.
12. It is OK to use violence if someone makes fun of you or insults you because of your religion, your origin or the color of your skin.
13. It is OK to use violence to solve the problems of the world.
14. It is OK to commit terrorist acts.
15. It is OK to use bombs to fight injustice.
